# Bi-ventricular assessment with cardiovascular magnetic resonance at 5 Tesla: A pilot study

**DOI:** 10.3389/fcvm.2022.913707

**Published:** 2022-09-12

**Authors:** Lu Lin, Peijun Liu, Gan Sun, Jian Wang, Dong Liang, Hairong Zheng, Zhengyu Jin, Yining Wang

**Affiliations:** ^1^State Key Laboratory of Complex Severe and Rare Diseases, Department of Radiology, Peking Union Medical College Hospital, Chinese Academy of Medical Sciences and Peking Union Medical College, Beijing, China; ^2^State Key Laboratory of Complex Severe and Rare Diseases, Department of Medical Science Research Center, Peking Union Medical College Hospital, Chinese Academy of Medical Sciences and Peking Union Medical College, Beijing, China; ^3^Lauterbur Research Center for Biomedical Imaging, Shenzhen Institute of Advanced Technology, China Academy of Sciences, Shenzhen, China

**Keywords:** 5T, UHF, cardiac MR, cine, ventricular function

## Abstract

**Background:**

Cardiovascular magnetic resonance (CMR) imaging at ultra-high fields (UHF) such as 7T has encountered many challenges such as faster T${}_{2}^{*}$ relaxation, stronger B_0_ and B_1+_ field inhomogeneities and additional safety concerns due to increased specific absorption rate (SAR) and peripheral nervous stimulation (PNS). Recently, a new line of 5T whole body MRI system has become available, and this study aims at evaluating the performance and benefits of this new UHF system for CMR imaging.

**Methods:**

Gradient echo (GRE) CINE imaging was performed on healthy volunteers at both 5 and 3T, and was compared to balanced steady-state-free-procession (bSSFP) CINE imaging at 3T as reference. Higher spatial resolution GRE CINE scans were additionally performed at 5T. All scans at both fields were performed with ECG-gating and breath-holding. Image quality was blindly evaluated by two radiologists, and the cardiac functional parameters (e.g., EDV/ESV/mass/EF) of the left and right ventricles were measured for statistical analyses using the Wilcoxon signed-rank test and Bland-Altman analysis.

**Results:**

Compared to 3T GRE CINE imaging, 5T GRE CINE imaging achieved comparable or improved image quality with significantly superior SNR and CNR, and it has also demonstrated excellent capability for high resolution (1.0 × 1.0 × 6.0 mm^3^) imaging. Functional assessments from 5T GRE CINE images were highly similar with the 3T bSSFP CINE reference.

**Conclusions:**

This pilot study has presented the initial evaluation of CMR CINE imaging at 5T UHF, which yielded superior image quality and accurate functional quantification when compared to 3T counterparts. Along with reliable ECG gating, the new 5T UHF system has the potential to achieve well-balanced performance for CMR applications.

## Introduction

As one of the frequently employed techniques in cardiac magnetic resonance imaging (CMR), cardiac CINE imaging enables accurate measurements of heart motion in a full cardiac cycle (1–4). Because CMR CINE imaging enables visualization and measurement of the periodic beating of the cardiac chambers and ventricular walls, it is routinely used for cardiac functional analysis (5, 6), such as chamber volume, myocardium muscle mass and blood ejection fraction (EF). Typically, clinical CINE images are collected either using 2D balanced steady-state-free-precession (bSSFP) or 2D spoiled gradient echo (GRE) sequences (7, 8).

To date, the majority of clinical CMR scans are performed at 1.5 and 3.0T. The studies to perform CMR at ultra-high fields (UHF), such as 7T, have been relatively limited (9–13). Generally speaking, UHF offers advantages of higher signal-to-noise ratio (SNR) and gradient performance for improved imaging workflow and quality (14). While such benefits also apply to CMR, CMR at UHF encounters many challenges that combinedly outweigh those benefits (15). The practical challenges include the shorter T_2_/T${}_{2}^{*}$ relaxation times, stronger B_0_ and B_1+_ inhomogeneities, higher specific absorption rate (SAR) of the radio frequency (RF) energy and stronger interference to the electrocardiogram for reliable cardiac gating, as well as the more stringent safety screening criteria (16, 17). Currently, CMR at UHF (≥7T) is at best considered as feasible or comparable to the 3T CMR (9, 13, 18).

Recently, a new 5T UHF MRI system was introduced for whole body imaging (19). At the midway between 3 and 7T, the field strength of 5T may have the potential to achieve a better balance between CMR performance/quality and those aforementioned challenges associated with UHF. In this work, we aimed to evaluate, for the first time, the feasibility and performance of CMR at the 5T whole body system. Specifically, we analyzed and compared CMR CINE results from 5T to those from 3T, both qualitatively and quantitatively.

## Materials and methods

### Subjects

Seventeen healthy adult volunteers (12 males and 5 females, age range 23–54) were recruited to participate in this pilot study with approval from the local ethics committee at Peking Union Medical College Hospital. All subjects passed the MRI safety screening with CMR-specific exclusion criteria, which strictly excluded candidates with prior or current cardiovascular diseases, severe arrhythmias, claustrophobia, metallic implants and tattoos, etc. All subjects provided written consents before undergoing CMR scans at both 3 (uMR790, United Imaging Healthcare, Shanghai, China) and 5T (uMR Jupiter, United Imaging Healthcare, Shanghai, China). Each volunteer underwent all MRI scans within the same day. The scanning order between 5T and 3T was randomized for each subject, with 7 subjects being scanned at 5T first.

### Experimental setups

The whole body 5T scanner was equipped with a bore size of 60 cm and a gradient system with 120 mT/m maximum amplitude and 200 T/m/s slew rate. For the RF system, an 8-channel loop array volumetric coil (19, 20) was used for transmission, and a 24-channel flexible body coil plus the upper portion of a 48-channel spine coil were used for reception during CMR scans. For B_1+_ inhomogeneity control within the field-of-view (FOV) of body scans, the complex B_1+_ map of each transmit channel was first obtained with a calibration scan and was then used to adjust the amplitude and phase of the respective transmit channel *via* parallel transmission techniques (21). Moreover, five 2nd order and one 3rd order shim coils were equipped for active B_0_ field shimming.

The 3T scanner has gradient system specifications of 100 mT/m maximum amplitude and 200 mT/m/ms slew rate, and the RF system was comprised of a dual-channel volumetric coil transmission coil and a 24-channel CMR-dedicated coil plus a portion of the spinal coil for signal reception. For B_0_ shimming, the volumetric shimming mode for cardiac scans was used.

**Figure 1:**
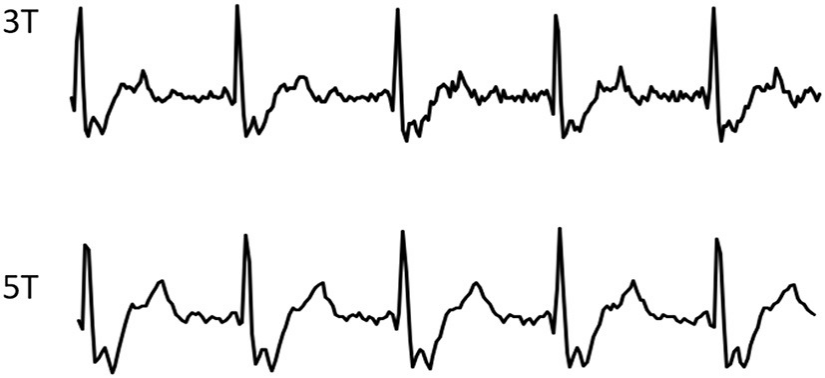
Typical ECG wave forms from one volunteer, recorded during CMR CINE scans at 3 and 5T respectively.

For all CINE scans at both fields, cardiac gating was performed using routine ECG with the 4-electrode placement scheme, the typical signals of which are shown in Figure 1.

### Imaging parameters

For 5T CMR scans, 2D GRE CINE images with a spatial resolution of 1.6 × 1.6 × 8.0 mm^3^ were acquired with the following parameters: TE/TR = 2.84/5.55 ms, flip angle (FA) = 10°, field of view (FOV) = 360 × 280~320 mm^2^, matrix size = 224 × 174~199, readout bandwidth = 300 Hz/px, 25 phases per cardiac cycle, and two-fold parallel imaging for acceleration, leading to a breath-hold duration of ~11 s per slice. Additionally, high resolution images (HR, 1.0 × 1.0 × 6.0 mm^3^) were also obtained using the same 2D GRE CINE sequence with increased matrix size of 352 × 274~313 and an additional 5~7 s (totaling 16~18 s) for breath-hold.

For 3T CMR scans, both 2D GRE CINE and 2D bSSFP CINE sequences were scanned with the same spatial resolution of 1.6 × 1.6 × 8.0 mm^3^. The GRE CINE protocol was: TE/TR = 2.65~2.69/5.42~5.45 ms, FA = 15°, readout bandwidth = 300 Hz/px, 25 phases per cardiac cycle, and two-fold parallel imaging for acceleration, leading to a breath-hold duration of ~11 s per slice. The bSSFP CINE protocol was: TE/TR = 1.37/2.98 ms, FA = 60°, readout bandwidth = 1,000 Hz/px, 25 phases per cardiac cycle, and two-fold parallel imaging for acceleration, leading to a breath-hold duration of ~6 s per slice. Other parameters were set the same as the 5T regular resolution protocol unless stated otherwise.

For all CMR scans, images of two-chamber (2 ch) and four-chamber (4 ch) views, as well as of a stack of short axis (SAX) view covering both left and right ventricles were obtained. Table 1 shows the summary of the scanning parameters. To achieve sufficient coverage for SAX scans, 10 slices with 20% gaps were acquired for regular scans, and 12 slices with 20% gaps for HR scans. Particularly, the TE of GRE scans were set to as close as possible to the nearest water-fat out-of-phase echo times (3T: 2.65 ms vs. the theoretical 2.20 ms; 5T: 2.84 or 3.04 ms vs. the theoretical 2.72 ms), to create a natural boundary between myocardium and fats for better tissue delineation in subsequent image analysis.

**Table 1:** CMR scanning parameters in this study.

	**3T bSSFP**	**3T GRE**	**5T GRE**	**5T GRE HR**
Short axis	Field of view (mm)	360 × 280~320	*360 × 280~320	360 × 280~320	360 × 280~320
	Spatial resolution (mm)	1.6 × 1.6	1.6 × 1.6	1.6 × 1.6	1.0 × 1.0
	Matrix	224 × 174~199	224 × 174~199	224 × 174~199	352 × 274~313
	Number of slices	10	10	10	12
	Slice thickness (mm)	8	8	8	6
	Slice gap (mm)	1.6	1.6	1.6	1.2
	TE/TR (ms)	1.37/2.98	2.65~2.69/5.42~5.45	2.84/5.55	3.06/5.95
	Flip angle (°)	60°	15°	10°	10°
	Bandwidth (Hz/pixel)	1,000	300	300	300
2 Chamber	Field of view (mm)	360 × 280~320	360 × 280~320	360 × 280~320	360 × 280~320
	Spatial resolution (mm)	1.6 × 1.6	1.6 × 1.6	1.6 × 1.6	1.0 × 1.0
	Matrix	224 × 174~199	224 × 174~199	224 × 174~199	352 × 274~313
	Number of slices	1	1	1	1
	Slice thickness (mm)	8	8	8	6
	TE/TR (ms)	1.37/2.98	2.65~2.69/5.42~5.45	2.42/4.48	3.06/5.95
	Flip angle (°)	60°	15°	10°	10°
	Bandwidth (Hz/pixel)	1,000	300	500	300
4 Chamber	Field of view (mm)	360 × 260~320	360 × 260~320	360 × 260~300	360 × 260~300
	Spatial resolution (mm)	1.6 × 1.6	1.6 × 1.6	1.6 × 1.6	1.0 × 1.0
	Matrix	224 × 162~187	224 × 162~187	224 × 162~187	352 × 254~293
	Number of slices	1	1	1	1
	Slice thickness (mm)	8	8	8	6
	TE/TR (ms)	1.37/2.98	2.65/5.42	2.42/4.48	3.06/5.95
	Flip angle (°)	60°	15°	10°	10°
	Bandwidth (Hz/pixel)	1,000	300	500	300

### Image analysis

All image analyses described below were performed by two experienced radiologists with 10 and 5 years of experience in CMR. The readers were blinded to the acquisition parameters, field strengths and subject demography, and they received all the CMR CINE images in a randomized order.

### Quality evaluation

Overall image quality (jointly on boundary sharpness, anatomic feature visibility and noise level) and the presence of artifacts (including those related to motion and reconstruction) were evaluated with qualitative scores from 0 to 3. The scoring criteria on image quality were defined as: 0, poor, non-diagnostic; 1, impaired image quality for potential misdiagnosis; 2, good quality meeting routine standards; and 3, excellent, exceeding routine quality. And the scoring criteria on artifacts were: 0, no artifact present; 1, mild artifacts not impairing diagnostic quality; 2, moderate artifacts partially impairing diagnostic quality; and 3, severe artifacts with non-diagnostic quality. Scores were independently given by the two radiologists on SAX and long-axis images of each subject. The Wilcoxon matched pairs test was used to compare the results.

### SNR-CNR analysis

For SNR and CNR assessment, regions of interest (ROI) signals were manually extracted from end-diastole SAX slices on the midventricular level (10). One ROI was positioned in the center of the left ventricle (LV) cavity, and 6 ROIs were drawn in the myocardium according to a six-segment model (22) and the mean result were reported. The noise level was estimated by drawing three background ROIs free from any visible artifacts and taking the average of the signal standard deviation (SD). The SNR was calculated by dividing the mean signal intensity of the LV cavity by the noise:


$$\begin{array}{l} \text{SNR}=\frac{S_{cavity}}{mean\left(SD\left(air\right)\right)} \end{array}$$

The CNR was estimated as the ratio of the mean signal difference between LV cavity and LV myocardium to the noise SD:


$$\text{CNR}=\frac{S_{cavity}-mean(S_{myocardium})}{mean(SD(air))}$$

The Wilcoxon matched pairs test was used to compare the results.

### Functional analysis

Quantitative functional measurements on end-diastolic volume (EDV), end-systolic volume (ESV), EF for both LV and right ventricle (RV) were assessed by the same radiologists using CVI42 (Circle Cardiovascular Imaging, Calgary, AB, Canada). The myocardium boundaries on SAX images were initialized using the software and manually adjusted when necessary. The myocardium mass of LV was also assessed, with papillary muscles and trabeculae excluded from the myocardium mass but included in the blood pool volume calculation.

Statistical analyses on the functional measurements were performed using Student's *t*-test after normality test. *P*-values of multiple comparisons were adjusted using Holm-Bonferroni method. A difference was considered significant with *p* < 0.05. Correlations among measurements were evaluated using Bland-Altman analysis.

## Results

Figure 1 shows the typical ECG signals recorded from both 5 and 3T during the CINE scans, both showing highly similar and reliable wave forms.

**Figure 2:**
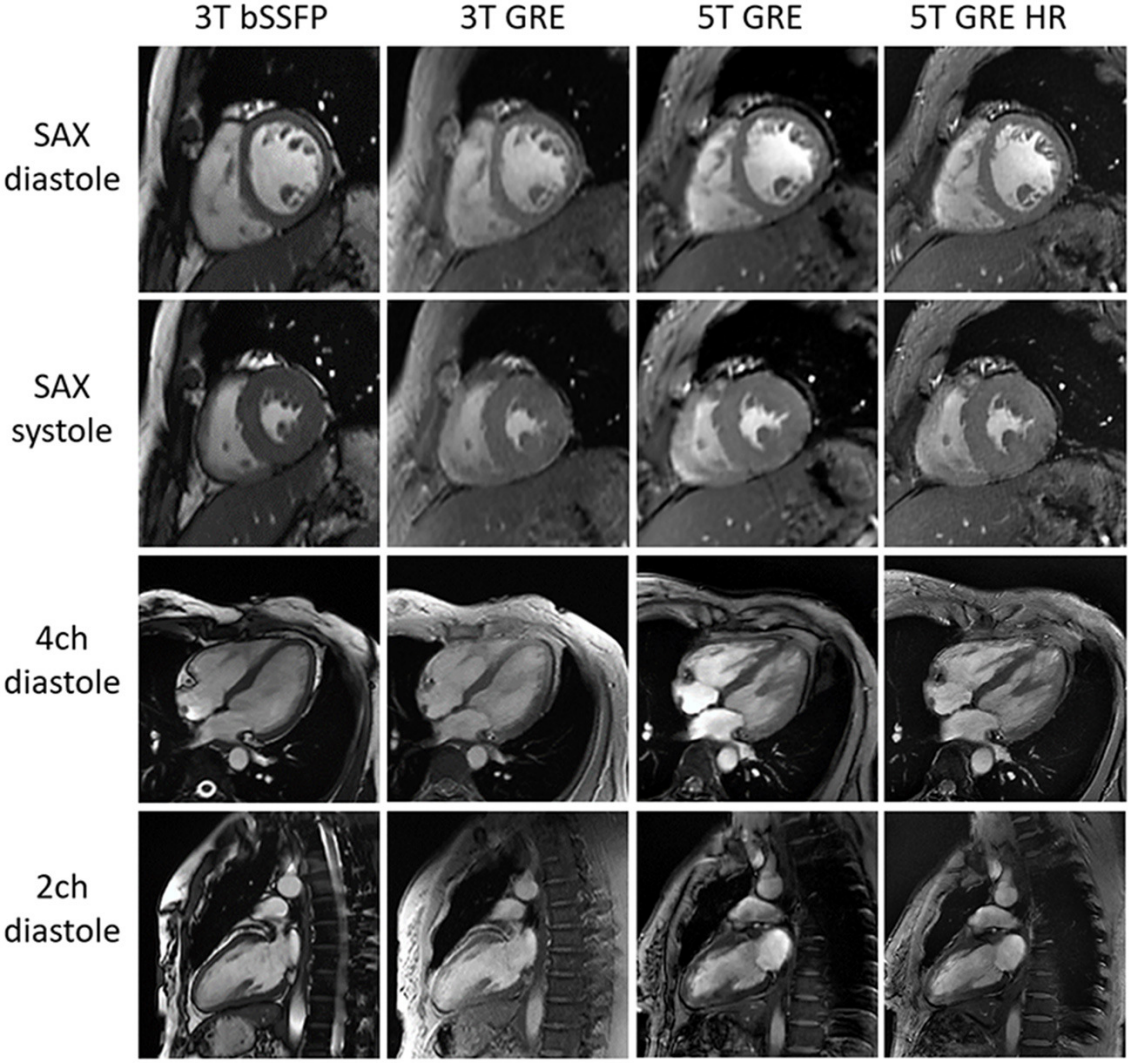
Typical CMR cine images of one subject. All images are free of major artifacts and show good image quality.

Figure 2 shows representative CINE images at both 5 and 3T, all displaying image quality that meets the routine clinical needs. Note that the fine structures of papillary muscles are clearly visible in the 5T GRE HR images.

**Figure 3:**
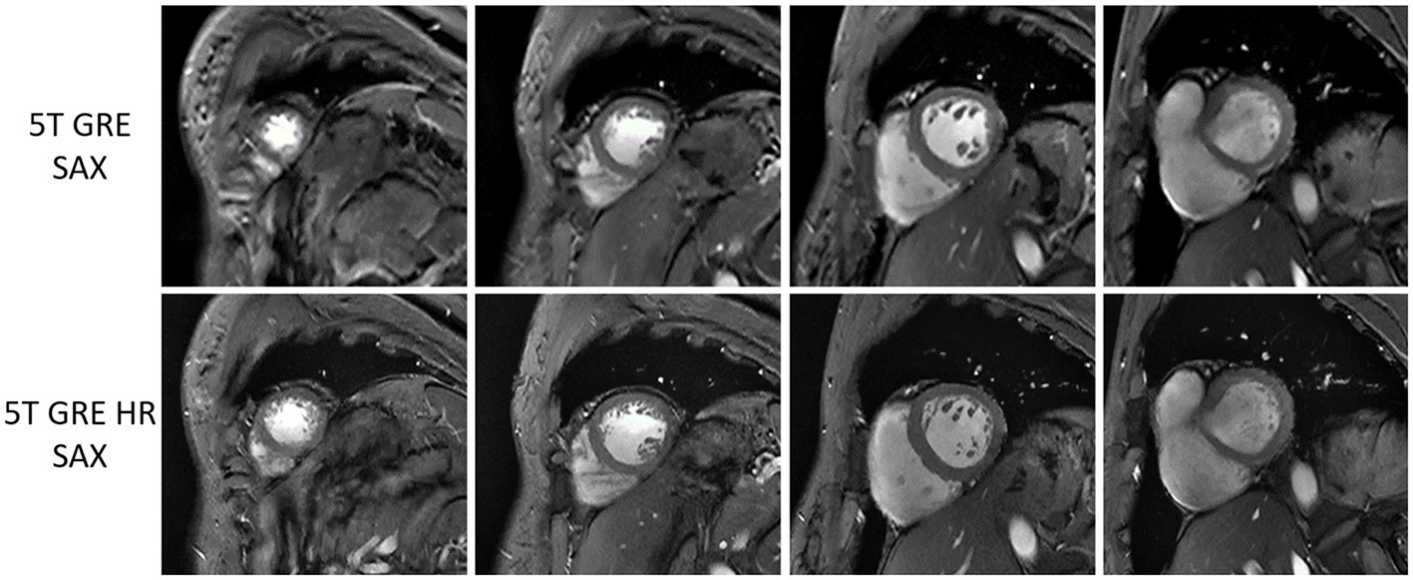
Multi-slice short-axis GRE images at 5T of one volunteer, comparing between routine resolution (top row) and high resolution (bottom row).

Figure 3 compares the 5T GRE and 5T GRE HR SAX images. While both scans yielded excellent image quality, image contrasts and SNR, the HR images enabled better delineation of finer structures such as myocardium-blood boundaries, papillary muscles and trabecular structures.

### Quality evaluation

The evaluation scores on image quality and artifact presence are summarized in Table 2. All GRE results, i.e., 3T GRE, 5T GRE, and 5T GRE HR, showed statistically similar scores in SAX images. For long axis images, 5T GRE, and 5T GRE HR images mostly received significantly better scores in both image quality and artifact control than 3T GRE, except the artifact control of 5T GRE being similar to 3T GRE (*p* = 0.07). 3T bSSFP showed significantly better scores than 3T GRE in all categories, as expected.

**Table 2:** Rating of overall image quality and presence of artifacts, using 3T GRE as reference.

**Slice orientation**	**Sequence**	**Voxel size (mm^3^)**	**Image quality (mean ±STD)**	**Artifacts rating (mean ±STD)**
SAX	3T GRE	1.6 × 1.6 × 8	2.0 ± 0.5	1.2 ± 0.4
	3T bSSFP	1.6 ×1.6 ×8	2.5 ± 0.6 (*p* = 0.014)*	0.6 ± 0.6 (*p* = 0.002)*
	5T GRE	1.6 ×1.6 ×8	2.2 ± 0.5 (*p* = 0.250)	1.2 ± 0.4 (*p* = 1.000)
	5T GRE HR	1.0 ×1.0 ×6	2.1 ± 0.8 (*p* = 0.424)	1.4 ± 0.5 (*p* = 0.219)
Long axis	3T GRE	1.6 ×1.6 ×8	1.5 ± 0.5	1.4 ± 0.6
	3T bSSFP	1.6 ×1.6 ×8	2.6 ± 0.5 (*p* <0.001)*	0.4 ± 0.5 (*p* <0.001)*
	5T GRE	1.6 ×1.6 ×8	1.9 ± 0.5 (*p* = 0.039)*	1.1 ± 0.5 (*p* = 0.070)
	5T GRE HR	1.0 ×1.0 ×6	2.3 ± 0.7 (*p* <0.001)*	1.1 ± 0.4 (*p* = 0.039)*

Both rating scores ranged from 0 to 3, and a higher rating denotes better image quality, but worse artifact control.

Asteroids indicate significant differences, i.e., *p* < 0.05, when compared to 3T GRE.

Table 3 shows the SNR of myocardium signals and CNR between myocardium and blood pool. In accordance to literatures (10), SNR of 3T bSSFP (130.9 ± 23.2) was significantly higher than 3T GRE (91.8 ± 10.0). On the other hand, the 5T GRE results, under the same routine resolution of 1.6 × 1.6 × 8 mm^3^, also achieved significantly higher myocardium SNR (1,438.0 ± 26.3) and CNR (92.5 ± 22.7) than 3T GRE images. With ~70% smaller voxel volume, the GRE HR images at 5T achieved lower SNR (62.5 ± 9.9) and CNR (37.4 ± 10.2) when compared to 3T GRE.

**Table 3:** Comparison on SNR and CNR of SAX images, using 3T GRE as reference.

**Sequence**	**Voxel size (mm^3^)**	**SNR (mean ±SD)**	**Relative SNR**	**CNR (mean ±SD)**	**Relative CNR**
3T GRE	1.6 ×1.6 ×8	91.8 ± 10.0	1.00	52.1 ± 13.7	1.00
3T bSSFP	1.6 ×1.6 ×8	130.9 ± 23.2 (*p* = 0.016)*	1.43	94.0 ± 21.5 (*p* = 0.007)*	1.80
5T GRE	1.6 ×1.6 ×8	143.8 ± 26.3 (*p* = 0.013)*	1.57	92.5 ± 22.7 (*p* = 0.034)*	1.78
5T GRE HR	1.0 ×1.0 ×6	62.5 ± 9.9 (*p* = 0.012)*	0.68	37.4 ± 10.2 (*p* = 0.145)	0.72

Asteroids indicate significant differences, i.e., *p* < 0.05, when compared to 3T GRE.

### Functional statistics

Table 4 compares all the functional parameters (i.e., LV EDV/ESV/Mass/EF and RV EDV/ESV/EF) among the 4 CINE image sets (i.e., 3T bSSFP/GRE and 5T GRE/GRE HR), using the 3T bSSFP results as reference. The consistency of all functional parameters are in good agreement with literatures (10, 12). Most functional measurements did not show statistically significant differences, except for RV EDV (all GRE), RV EF (3T GRE), and LV Mass (5T GRE).

**Table 4:** Comparison of CMR CINE functional quantities, using 3T bSSFP as reference.

**Sequence**	**LV EDV (mm^3^)**	**LV ESV (mm^3^)**	**LV Mass (g)**	**LV EF (%)**	**RV EDV (mm^3^)**	**RV ESV (mm^3^)**	**RV EF (%)**
3T bSSFP	129.8 ± 18.5	45.1 ± 9.1	98.1 ± 21.2	65.3 ± 4.1	142.2 ± 20.5	63.0 ± 13.4	56.1 ± 4.7
3T GRE	128.1 ± 20.2	43.3 ± 9.2	105.7 ± 20.7	66.4 ± 5.0	148.7 ± 21.4*	62.3 ± 13.1	58.4 ± 4.9*
5T GRE	130.1 ± 19.2	44.0 ± 11.6	104.0 ± 20.2*	66.8 ± 6.0	152.1 ± 21.3*	66.5 ± 12.7	57.4 ± 4.7
5T GRE HR	129.6 ± 18.0	45.2 ± 10.8	101.5 ± 19.5	65.4 ± 5.2	151.5 ± 20.2*	65.8 ± 13.8	56.8 ± 6.5

Asteroids indicate significant differences, i.e., *p* < 0.05, when compared to 3T bSSFP.

**Figure 4:**
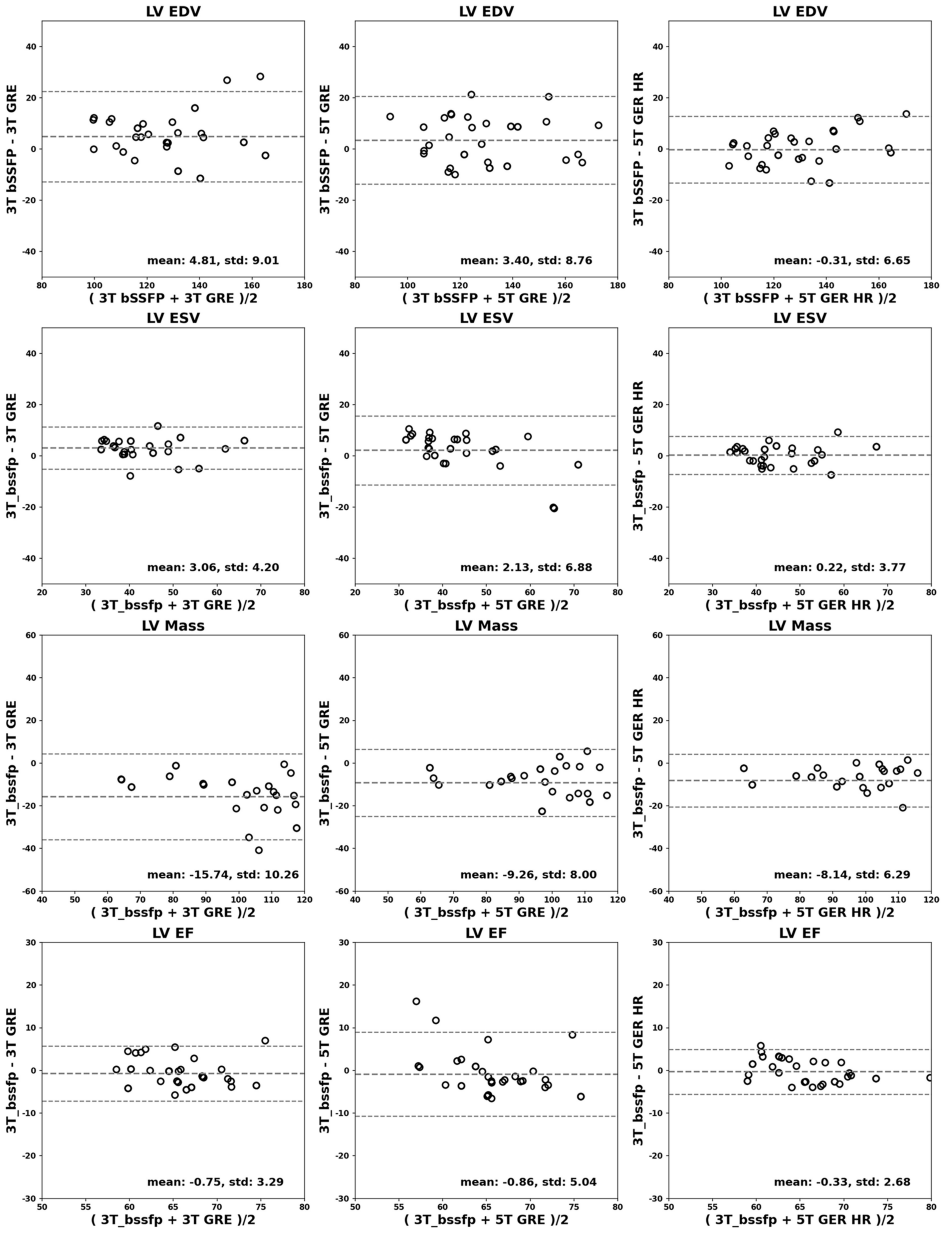
Bland-Altman analyses for LV. LV EDV (ml), ESV (ml), mass (g) and EF (%) are shown in rows. Comparisons of 3T GRE, 5T GRE, and 5T GRE HR to 3T bSSFP are shown in columns.

**Figure 5:**
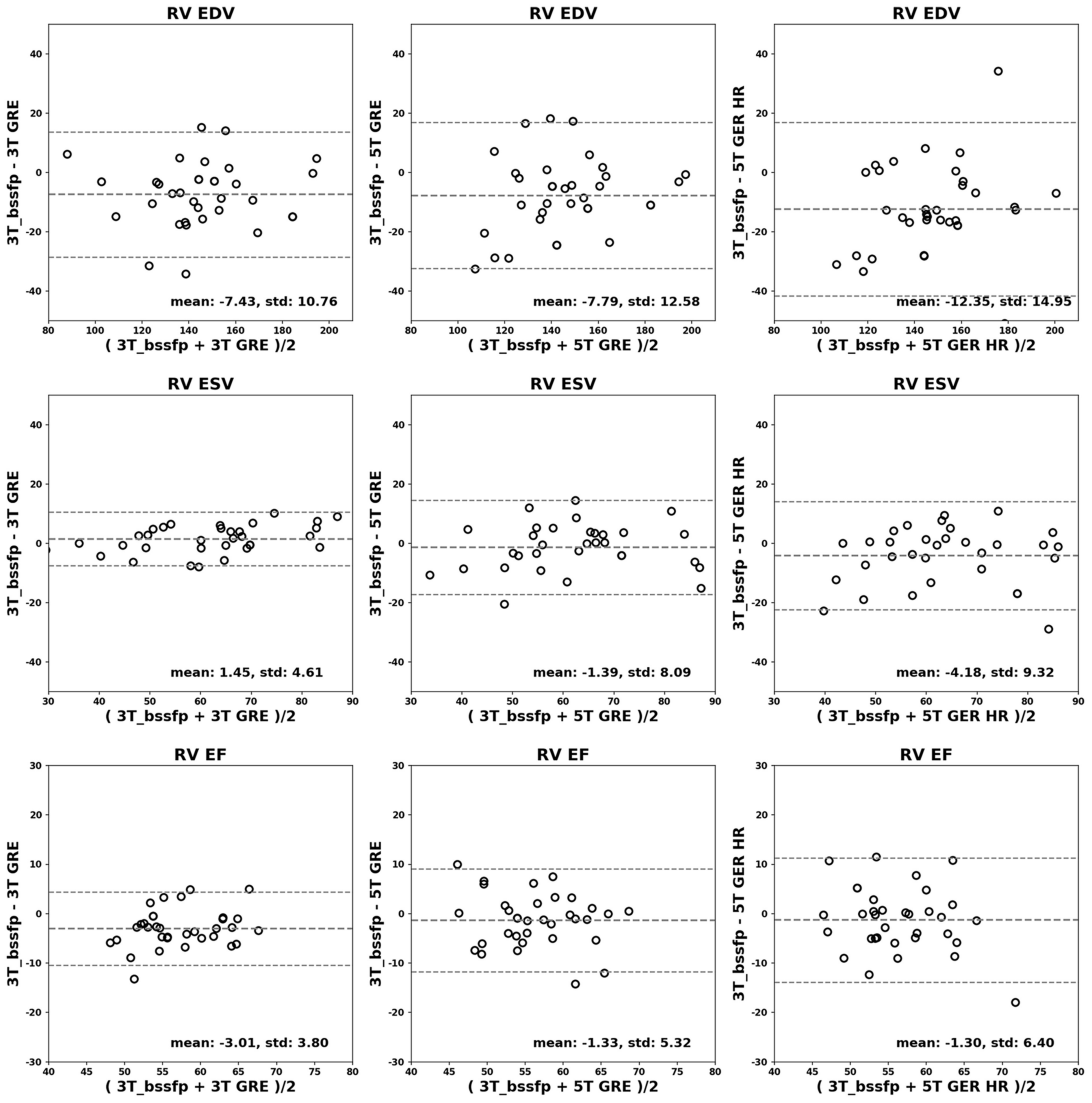
Bland-Altman analyses for RV. RV EDV (ml), ESV (ml) and EF (%) are shown in rows. Comparisons of 3T GRE, 5T GRE, and 5T GRE HR to 3T bSSFP are shown in columns.

Figures 4, 5 show the Bland-Altman plots for cardiac functional analyses on LV and RV, respectively. Both figures indicate good agreement of all GRE scans including 5T's to the reference 3T bSSFP scans.

## Discussion

In this pilot study, we performed an initial evaluation of CMR performance on a newly developed 5T whole body system (19). We used the 3T GRE CINE images as reference for image quality and SNR comparison, and used the 3T bSSFP images for the functional assessment accuracy comparison (23). The results not only demonstrated the feasibility of CMR imaging at 5T, but also suggested that 5T CMR may provide additional benefits compared to routine high field (e.g., 3T) by offering improved and balanced outcomes.

The 5T system provided excellent image quality and contrasts between myocardial muscles and blood, showing improved contrasts than the 3T GRE images. There are several factors that may contribute to such improvement in image quality.

First, the higher field creates a stronger time-of-flight inflow effect on the inflowing blood (10), thus increasing the myocardium-blood contrast and leading to visually sharper boundaries, even under the same resolution settings. This is true for all slice orientation settings (i.e., SAX, 4 ch, and 2 ch views), as evidenced in Figure 2. Such improved tissue contrast potentially offers more useful details for applications involving cardiac morphology (24), such as arrhythmogenic right ventricular dysplasia (ARVD) (25) where the trabeculae carneae may be obscured within relatively slower (than LV) flowing blood.

Second, the gain in image quality at 5T was also evidenced by the increase in SNR and CNR. With regular resolution of 1.6 × 1.6 × 8.0 mm^3^, the 5T GRE images showed significantly superior SNR and CNR than the GRE at 3T. Even with a much smaller voxel size (~30% in voxel volume), the 5T GRE HR images still yielded sufficient SNR and CNR to support similar or even better visual quality than the routine 3T GRE. Unlike in most 7T CMR studies where local TX/RX coils (9, 11, 12, 18) were used, the 5T system employed a volumetric body coil for parallel RF transmission. With an 8-channel loop array design, the B_1+_ field can be dynamically adjusted on a subject-by-subject basis. The advantage of using such a “global” parallel transmission over the “local” transceiver is that it can achieve more homogeneous excitation effects, especially on patients with larger girth sizes.

Third, the 5T system is capable of detecting almost distortion-free ECG signals for reliable gating during CMR scans, highly comparable to those from 3T (Figure 1) and much better than those from 7T (26). ECG signals originate from the cardiac electrical activities associated with the cardiac rhythmic motions, which is field independent by itself. However, electrode-based ECG detection in MRI is usually overlaid with the so-called magneto-hydrodynamic (MHD) effects (27) originating from the conducting fluid (i.e., cardiac blood flow) under the influence of the external magnetic field and will become stronger at higher fields. Practically, the MHD effect is negligible at 3T and lower fields, but it has been determined to severely affect ECG readings at 7T (26) to the extent of non-usability. Therefore, alternative ECG gating techniques have been generally needed at UHF, such as acoustic cardiac triggering (28) or the finger-clipping pulse oximetry. However, these gating methods only provide an alternative form of the true ECG signals and have their own limitations (28). During our scanning process at 5T, the ECG quality of all subjects, both males and females, was sufficiently reliable to yield high quality images with minimal motion artifacts, while requiring no additional efforts than at 3T.

Finally, the combination of the high SNR and reliable ECG gating at 5T supports reliable high-resolution CMR scans. In this study, the high-resolution was set as 1.0 × 1.0 × 6.0 mm^3^, which is <30% in voxel volume comparing to routine scan settings and has been rarely reported at 3T due to compromise in SNR. Nevertheless, the 5T GRE HR images still achieved satisfying SNR and CNR, suggesting the SNR benefit at UHF was indeed well utilized at 5T. On the other hand, the HR scans required a longer scan time by 5~7 s, a reliable ECG gating is critical to containing the risks of motion artifacts. As revealed in the image results and visual rating statistics (Figure 2 and Table 2), the 5T GRE HR scans showed comparable or even better artifact control than 3T GRE, also demonstrating the traditional ECG method is well employed at 5T.

To briefly summarize, the improvement in imaging contrast and SNR/CNR, as well as the robust ECG gating capacity suggest the potential of the 5T UHF for reliable CMR imaging on cardiovascular anatomy and functionalities. Although 5T is midway between 3 and 7T, the 5T CMR setup, in terms of scanning parameters and the ECG gating scheme, was more similar to those at 3T, while its performance on SNR/CNR and imaging quality may be more 7T alike. Other challenges reported at 7T, such as B_1+_ field inhomogeneity and high SAR levels, were also observed at 5T but with much milder effects, thus are expected to be coped with more easily.

Regarding the cardiac functional results, all scans from both systems yielded statistically comparable results, with only a few exceptions (Table 4). This was somewhat different from previous reports, where significant differences were observed for LV EDV/ESV/EF and RV EDV/ESV between bSSFP and GRE at 3T (5), or for LV mass between 7 (both regular- and high-resolution) and 1.5T GRE (10). The cause for such differences was previously attributed to the epicardial fat being differentiable in bSSFP images but not in GRE images (10), thus leading to large myocardium ROIs drawn on GRE images by including fatty tissues. In this study, by using out-of-phase echo times for GRE scans, visible myocardium-fat boundaries were created, which was clearly shown in all our pictorial figures. Therefore, with the automatic myocardium extraction by the CVI42 software followed by manual ROI adjustment, the cardiac functional quantities can be more reliably extracted even from GRE data, avoiding over-estimation by excluding most of the fatty tissues.

Several limitations of this study should be noted. In this pilot study, only a limited number of healthy volunteers without any cardiac conditions were included in the functional and statistical analyses. As has been demonstrated in a previous study (12) that HR CINE imaging at 7T can reveal myocardial crypts in hypertrophic cardiomyopathy patients, which were obscured at 3T. The benefits of imaging with better contrasts, SNR and resolution at 5T are yet to be demonstrated with a larger patient cohort with cardiovascular diseases. Second, this work only focused on CINE imaging, and other CMR techniques such as tissue characterization (29) and quantitative mapping (30, 31), should be further evaluated in future works. Third, only a normal flexible body coil was currently available on the 5T system, while further improvement in imaging quality can be expected with dedicated cardiac coils (32).

Since the 5T MRI system has become available only very recently, virtually any CMR techniques and applications can be revisited. Judging from the results of this study and from literatures, it is likely that CMR imaging at 5T can enjoy more of the pros from both sides while suffering from less of the cons. For instance, it is worth mentioning that in our preliminary tests on 5T bSSFP CINE scans, the SAR level (<13 W/kg) was well within the first level safety limit (i.e., 20 W/kg for body trunk), although the robustness of imaging quality against B_0_ and B_1_ effects still required improvement (thus data not shown). Since bSSFP CINE is generally considered the standard method (compared to GRE CINE) at 1.5 and 3T fields but is well-known to be unusable at 7T UHF (15, 17), its potential feasibility at 5T UHF will be highly desirable.

Moreover, with the stronger T_1_ sensitivities, 5T may foreseeably have better performance on related applications such as quantitative perfusion or late gadolinium enhancement imaging, which can be of great interests for future works.

## Conclusion

In conclusion, this pilot study has presented the initial evaluation of CMR CINE imaging at 5T UHF. The image quality at 5T was generally superior or comparable to the 3T counterparts, while functional quantification results were statistically similar. Therefore, the new 5T UHF system has the potential to achieve a well-balanced performance for CMR CINE imaging to potentially meet the clinical and researching needs.

## Data availability statement

The raw data supporting the conclusions of this article will be made available by the authors, without undue reservation.

## Ethics statement

The studies involving human participants were reviewed and approved by the Institutional Review Board of Peking Union Medical College Hospital, Beijing, China. The patients/participants provided their written informed consent to participate in this study.

## Author contributions

LL and PL proposed the project and experimental designs and performed the subjective evaluation on imaging quality scoring and the post processing for subsequent quantitative analysis. YW, ZJ, DL, and HZ were involved in study set-up, interpretation of results, scientific, and clinical consultation. GS performed data acquisition and preparation. JW performed the statistical analysis. The manuscript was mainly prepared by LL and PL, with input and improvement from all other authors. All authors have read and approved the final manuscript.

## Funding

This work was partially supported by the National Key R&D Program of China (2017YFC0108800), the Major International (Regional) Joint Research Project of the National Natural Science Foundation of China (82020108018), and the National Natural Science Foundation of China (81873891).

## Conflict of interest

The authors declare that the research was conducted in the absence of any commercial or financial relationships that could be construed as a potential conflict of interest.

## Publisher's note

All claims expressed in this article are solely those of the authors and do not necessarily represent those of their affiliated organizations, or those of the publisher, the editors and the reviewers. Any product that may be evaluated in this article, or claim that may be made by its manufacturer, is not guaranteed or endorsed by the publisher.

## Abbreviations

ARVD: arrhythmogenic right ventricular dysplasia
bSSFP: balanced steady state free precession
CMR: cardiac magnetic resonance
CINE: cinematic imaging
CNR/SNR: contrast/signal-to-noise ration
ECG: electrocardiogram
EDV: end diastolic volume
EF: ejection fraction
ESV: end-systolic volume
FOV: field-of-view
GRE: gradient echo
LV/RV: left/right ventricle
MHD: magneto-hydrodynamic
RF: radiofrequency
ROI: region of interest
SAR: specific absorption rate
SAX: stack of short axis
STD: standard deviation
UHF: ultra-high fields.
